# Conformational and functional analysis of molecular dynamics trajectories by Self-Organising Maps

**DOI:** 10.1186/1471-2105-12-158

**Published:** 2011-05-14

**Authors:** Domenico Fraccalvieri, Alessandro Pandini, Fabio Stella, Laura Bonati

**Affiliations:** 1 Dipartimento di Scienze dell'Ambiente e del Territorio, Università degli Studi di Milano-Bicocca, Piazza della Scienza 1, 20126 Milano, Italy; 2 Division of Mathematical Biology, MRC National Institute for Medical Research, The Ridgeway, London NW7 1AA, UK; 3 Dipartimento di Informatica Sistemistica e Comunicazione, Università degli Studi di Milano-Bicocca, Viale Sarca 336, 20126 Milano, Italy

## Abstract

**Background:**

Molecular dynamics (MD) simulations are powerful tools to investigate the conformational dynamics of proteins that is often a critical element of their function. Identification of functionally relevant conformations is generally done clustering the large ensemble of structures that are generated. Recently, Self-Organising Maps (SOMs) were reported performing more accurately and providing more consistent results than traditional clustering algorithms in various data mining problems. We present a novel strategy to analyse and compare conformational ensembles of protein domains using a two-level approach that combines SOMs and hierarchical clustering.

**Results:**

The conformational dynamics of the α-spectrin SH3 protein domain and six single mutants were analysed by MD simulations. The Cα's Cartesian coordinates of conformations sampled in the essential space were used as input data vectors for SOM training, then complete linkage clustering was performed on the SOM prototype vectors. A specific protocol to optimize a SOM for structural ensembles was proposed: the optimal SOM was selected by means of a Taguchi experimental design plan applied to different data sets, and the optimal sampling rate of the MD trajectory was selected. The proposed two-level approach was applied to single trajectories of the SH3 domain independently as well as to groups of them at the same time. The results demonstrated the potential of this approach in the analysis of large ensembles of molecular structures: the possibility of producing a topological mapping of the conformational space in a simple 2D visualisation, as well as of effectively highlighting differences in the conformational dynamics directly related to biological functions.

**Conclusions:**

The use of a two-level approach combining SOMs and hierarchical clustering for conformational analysis of structural ensembles of proteins was proposed. It can easily be extended to other study cases and to conformational ensembles from other sources.

## Background

Protein dynamics plays a central role in cell life. In many cases biological function involves molecular motion [1] and it was recently suggested that intrinsic dynamics also defines the ability of proteins to adapt and evolve new functions [2]. Therefore, a full understanding of protein function and evolution will require a deeper insight into biomolecular atomistic dynamics.

Significant contributions in this direction have come from computational methods, in particular from Molecular dynamics (MD) simulations [3,4], by which a large ensemble of molecular structures can be generated to sample the accessible conformational space of a protein. Analysis of this ensemble can provide information about average physico-chemical and geometrical properties, as well as allowing identification of recurring conformations and transitions between them.

As recurrence of and transition between conformations is difficult to extract from the raw ensemble data [5], grouping the conformations becomes a necessity. The most desirable strategy would be to use *kinetic clustering *[6-11] where conformations are grouped according to their transition probabilities during the simulation and identified clusters are directly related to the free energy landscape. A limitation to this approach arises from the need of an exhaustive sampling with convergence of all pair-wise transition probabilities [11]. A more affordable solution is to use *geometrical clustering*, because only a representative sampling of the accessible conformations is required. The underlying assumption is that structurally similar conformations lie in the same basin of the free energy surface. While often this is an acceptable approximation, a recent study has suggested caution in interpreting the clustering results [11].

Geometrical clustering for conformational analysis was introduced when simulation time increased up to nanoseconds generating tens of thousands of structures [12-14] and has been extensively used since then [5,11]. Several data-mining algorithms have been adopted but according to a recent survey [5] no general strategy is available: clustering results are often influenced by the type of algorithm and the choice of optimal parameters is mostly left to user experience and the specific case. Originally adapted to analyse protein folding simulations [12], these algorithms have mainly been implemented for multiple trajectories of the same system. However, recently a great interest has emerged in the comparison of protein flexibility of functionally related proteins as well as in studying the evolutionary conservation and specialization of protein dynamics across distant homologous proteins [2,15-21]. This new interest emphasises the need for more advanced tools to compare conformational ensembles of different protein domains especially when derived from extensive MD simulations [15,16,18,20].

Among the data-mining algorithms, *Self-Organising Maps *(SOMs) [22] were recently applied to conformational analysis of lipid molecules [23,24] and automatic clustering of protein-ligand docking poses [25]. In the context of MD analysis, a recent study [5] compared several methods, including SOMs and traditional clustering algorithms. While the SOMs were identified among the best performing methods, no algorithm emerged as the optimal solution.

In this contribution, we present the use of a two-level approach [26] combining SOMs and hierarchical clustering [27] for the analysis and comparison of multiple MD trajectories of a protein domain. First we illustrate the protocol we developed to characterize and optimize the parameters of the SOM learning process for structural ensembles. Then we present the application of the proposed two-level approach, with the optimized parameters, to the conformational and functional analysis of a test case composed of the α-spectrin SH3 (Spc-SH3) protein domain and a group of its single-site mutants. This is an interesting study case of a small intra-cellular signaling domain [28] where ligand binding activity is modulated by single-mutations greatly affecting the conformational dynamics [29,30]. This test demonstrated the potential of the proposed approach in the analysis of large ensembles of molecular structures: the possibility of producing a topological mapping of the conformational space embedded in a simple 2D visualisation, given by the SOM stage, as well as the capability of effectively highlighting differences in the conformational dynamics directly related to biological functions, given by the combination of SOMs and the post-clustering stage.

## Methods

### Conformational sampling and ensemble generation

#### Molecular Dynamics Simulations

The atomistic dynamics of the set of representative proteins was simulated using the GROMACS package (version 3.3.3) [31-33] with the GROMOS96 43a2 force field. All structures were inserted into an octahedral box with explicit solvent and simulated with periodic boundary conditions. Water molecules were described by a simple point charge (SPC) model [34] and the box size was set to ensure a distance of at least 1.2 nm between the protein and the box boundaries. The solvent was relaxed with a 5 ps MD simulation, then the systems were neutralized by insertion of counter ions, and a short minimization with steepest descent was performed up to convergence on maximum force lower than 1000 kJ/(mol*nm). The resulting systems were simulated for 40 ns in the NPT ensemble (constant number of atoms N, pressure P, and temperature T). Long-range electrostatic interactions were calculated with the particle mesh Ewald (PME) summation method [35,36], with a 9 Å cut-off for the direct space s ums, a 1.2 Å FFT grid spacing and a 4-order interpolation polynomial for the reciprocal space sums. A thermal bath was independently coupled with protein and solvent using a Berendsen thermostat at 300 K with coupling period of 0.1 ps. The internal degrees of freedom of water were constrained by the Settle algorithm [37], while all bond distances in the protein were constrained by the LINCS algorithm [38]. The integration step was set to 2 fs.

To evaluate the sampling efficiency the overlap between the conformational spaces spanned by different parts of the simulation [39,40] was calculated. In general, the overlap between two matrices A and B, s(A,B), can be defined as:(E1)

where *tr *is the trace of the matrix. When *s(A,B) *is equal to 1, the two spanned subspaces are identical, whereas a value of 0 indicates complete orthogonality. In this work, the overlap between the covariance matrix of the atomic coordinates in each half of a simulation and in the overall trajectory [40] was evaluated for each protein.

#### Analysis of conformational flexibility

Protein flexibility was calculated on a residue basis using the root mean square fluctuation (RMSF) of the positions of the Cα atoms. The RMSF of the Cα atom *i *measures its deviation from the time-averaged position during the simulation:(E2)

where ***r***_*i *_is the coordinate vector for atom *i *and the < > indicates a time-averaging. To increase the signal/noise ratio, the RMSF calculation was performed after Essential Dynamics (ED) analysis. ED is a widely applied technique based on principal component analysis (PCA) of conformational ensembles [41], aimed to extract informative directions of motion. Only Cα atoms were included in the analysis [41].

The geometry of the protein binding site was described by a selected set of pair-wise atomic distances and its conformational changes were measured by distance Root Mean Square Deviation (dRMSD), *i.e*. the RMSD between these atomic distances in a selected conformation, *a*, and the same distances in a reference structure, *b*:(E3)

where *d *is the distance value, *i *and *j *are the indices of the selected atoms and *N *is the total number of computed distances.

### Optimization of the Self-Organising Maps for structural ensemble analysis

In the proposed approach the SOM input data are large ensembles of conformations extracted from the MD trajectories of one or more domains. Each conformation is described by the Cartesian coordinates of the Cα atoms of the protein, after projection of the original data on the eigenvectors that define the Essential Space. At the end of the SOM training process (see "Brief introduction to SOMs" for details) the original conformational ensemble ends up projected on a bi-dimensional feature map where similar elements are associated to neighbour neurons. Therefore the whole input space is described by a limited number of vectors (the prototype vectors of the neurons). These are then submitted to linkage clustering and the representative conformations are extracted for conformational and functional analysis.

A optimization of the SOM parameters for the specific type of data is required. The optimization we designed and performed consists of two steps: first, the SOM parameters were characterized and optimized by experimental design to obtain the optimal SOM and then these were used to select the optimal sampling rate of the MD trajectory.

**Figure 1:**
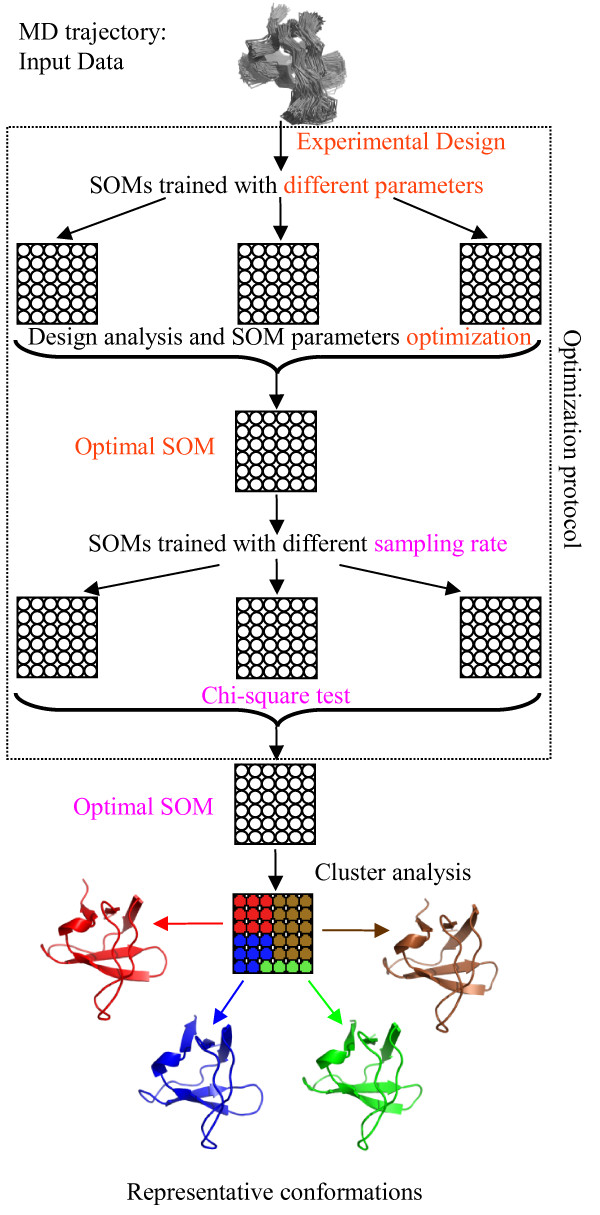
**Two-level approach for structural ensemble analysis**. Diagram of the proposed approach for structural ensemble analysis, including the optimization of the Self-Organizing Map performed in this work. Optimization protocol: the **Experimental Design **allows the training of SOMs with different parameters (**SOMs trained with different parameters**); the output of the SOMs training is used to optimize the SOM parameters (**Design analysis and SOM parameters optimization**) and to find the **Optimal SOM **which is trained with different sampling rates (**SOMs trained with different sampling rates**); the output of this training process is submitted to a **Chi-square test **to confirm the **Optimal SOM**. Two-level approach: the prototype vectors of the optimal SOM trained with the MD conformational ensemble are submitted to a clustering process (**Cluster analysis**) which extracts the **Representative conformations**.

A diagram of the overall approach, including the optimization steps performed in this work, is shown in Figure 1. In the following we report: a brief conceptual introduction to SOMs, a detailed description of each step of our optimization protocol, and the approach we propose for clustering.

#### Brief introduction to SOMs

A SOM is a powerful tool for the grouping and visualization of high-dimensional data. It is a specific architecture of artificial neural networks consisting of a low-dimensional (usually bi-dimensional) grid of so-called neurons, called map [42]. The number of neurons may vary from few units up to thousands and each neuron is represented by a *d*-dimensional model vector (the *prototype vector*), where *d *is the dimension of the input data vectors (in this application *d *is the number of Cα coordinates). The neurons are connected to adjacent neurons by a neighborhood relation, which dictates the topology of the SOM map.

The SOM learning algorithm could be divided into five steps: a) initial randomization of each prototype vector; b) random selection of an input vector; c) neurons "competition" to "win" the input vector; d) update of the prototype vector of the "winning" neuron and of its topological neighbors by pulling them closer to the "won" input vector; e) repeat from b) for each input vector for a given number of epochs sufficient to ensure a convergent training of the map. As a result, the prototype vector of each output neuron represents a particular feature (conformation) drawn from the input space [42].

A useful tool to analyse the results is a pictorial representation of the trained map. In this work we choose a topographic map where each neuron is represented by an hexagon. To visualize the number of input data won by each neuron (*hits*), black hexagons with size proportional to the neuron population were superimposed to the map. Visual inspection of the map allows the analysis of the final distribution of the original data: neighbor neurons (hexagons) are associated to similar groups of input data (conformations); map regions with highly-populated neurons represent conformations highly sampled during the trajectory.

The main interpretational advantage over hierarchical clustering is that maps are more easily understood and interpreted by humans than dendrograms, which become already very complex in the case of a few hundred data points.

In this work, the analysis was performed using the SOM toolbox in MATLAB [43], but in principle it can be reproduced using other statistical packages, such as R [44]. The calculations were performed on an AMD Opteron 254 1.8 GHz.

#### Experimental design and map optimization

The result of a SOM learning process depends on a set of parameters and the problem of finding the optimal SOM, i.e. the SOM model which "best characterizes" the underlying input space structure, can be extremely complex. The goal is first to discover the parameters that significantly affect the performance of the SOM model, and then to find the values of such parameters that define the optimal SOM. Unfortunately this is an ill-posed optimization problem [45], so several efforts have been made to provide an efficient and effective solution [46,47].

In this work, to reliably represent how well the SOM model captures the relevant features of the input space, the following performance measure, i.e. index of clustering efficiency, was used:(E4)

where *d *represents Euclidean distance, *j *and *C*_j _are the *j^th ^*cluster of neurons and the corresponding set of neurons, ***w***_*ij *_is the weight vector associated with the *i^th ^*neuron of the *j^th ^*cluster, ***μ***_*j *_and ***μ ***are the centroid of the *j^th ^*cluster and of the overall map. The performance measure (E4) is a modification of the clustering efficiency index described in [48] where  measures the average cluster tightness while measures the separation of centroids. However, the term  scales with the number of output neurons and thus it was normalized through *R_tot _*to account for different SOM sizes. The performance measure (E4) has a unique minimum and was successful against expert-based assessment of clustering [48].

The problem of finding the optimal value of the performance measure is computationally expensive and Artificial Neural Networks are generally affected by the curse of dimensionality. This problem can be solved following different strategies [49], among which *Design of Experiments *or Experimental Design [50,51] was selected in this study. In this technique the experiments are designed to have rational relationships to the purposes, needs and physical limitations of the problem. Variables and their values are tested in combinations that ensure to answer the questions of interest as clearly and efficiently as possible. This offers advantages in the economy of the experimentation and provides straightforward estimates of experimental effects and variance [52]. In the present study a set of SOM models with different values of the parameters were selected according to an experimental design plan and the output was used to identify the SOM's most influential parameters and thus to select the optimal SOM model (see Figure 1).

Consistent with previous reports [43] the following SOM parameters were optimized: *map size*, *learning algorithm*, *neighbourhood function*, *lattice type*, *shape*, *alpha type*, *radius*, *training length *and *initial value of the parameter alpha *(parameter names as defined in MATLAB). The ranges of tested values are reported in Table 1. A commonly used rule to select the *map size *is based on the dimension of the input data vectors and an estimate of the training data variance (i.e. the ratio between the two largest eigenvalues of the training data matrix) [43]. Instead of testing only this value (200 for this data set), a range of *map sizes *was considered for the optimization (100 - 400).

**Table 1:** SOM parameters together with allowed ranges.

SOM parameter	Range
Map size	[100,400]
Lattice type	{hexagonal, rectangular}
Shape	{sheet, cylinder, toroid}
Learning algorithm	{batch, sequential}
Neighbour function	{gaussian, bubble, ep}
Alpha type	{inverse, linear, power}
Radius	{1, 2, 3}
Training length	[1000,5000]
Starting alpha	[0.01, 0.09]

*Map size *(number of neurons), *Lattice type*, and *Shape *define the topological characteristics of the map. The other parameters are associated to the learning process: *Learning algorithm*, *Neighbour function, Alpha type, Radius *(neighbourhoods' kernel), *Training length *(number of learning cycles or epochs), *Starting alpha *(parameter defining the initial learning rate).

The particular nature of the parameters does not allow the use of either the widely used *D-Optimality *or *G-Optimality *design criteria. Instead, a Taguchi robust design plan [53] was used, including 36 runs with 3 replicas each. The design plan is reported in Table 2.

**Table 2:** Taguchi design plan.

Map size	Lattice type	Shape	Learning algorithm	Neighbour function	Alpha type	Radius	Training length	Starting alpha	Time (s)
100	hexagonal	sheet	batch	Gaussian	inverse	2	1000	0.01	20
225	hexagonal	cylinder	batch	Bubble	power	3	3000	0.05	125
400	hexagonal	toroid	batch	Ep	linear	1	5000	0.09	450
100	hexagonal	sheet	sequential	Gaussian	linear	3	1000	0.09	270
225	hexagonal	cylinder	sequential	Bubble	inverse	1	3000	0.01	820
400	hexagonal	toroid	sequential	Ep	power	2	5000	0.05	12000
100	rectangular	cylinder	sequential	Gaussian	linear	1	5000	0.05	1350
225	rectangular	toroid	sequential	Bubble	inverse	2	1000	0.09	600
400	rectangular	sheet	sequential	Ep	power	3	3000	0.01	7700
100	rectangular	toroid	batch	Gaussian	power	1	3000	0.01	55
225	rectangular	sheet	batch	Bubble	linear	2	5000	0.05	205
400	rectangular	cylinder	batch	Ep	inverse	3	1000	0.09	95
100	hexagonal	toroid	sequential	Bubble	power	1	1000	0.05	290
225	hexagonal	sheet	sequential	Ep	linear	2	3000	0.09	1800
400	hexagonal	cylinder	sequential	Gaussian	inverse	3	5000	0.01	12800
100	rectangular	toroid	sequential	Bubble	inverse	3	3000	0.09	600
225	rectangular	sheet	sequential	Ep	power	1	5000	0.01	2500
400	rectangular	cylinder	sequential	Gaussian	linear	2	1000	0.05	2800
100	rectangular	sheet	sequential	Bubble	linear	3	5000	0.01	1400
225	rectangular	cylinder	sequential	Ep	inv	1	1000	0.05	650
400	rectangular	toroid	sequential	Gaussian	power	2	3000	0.09	8500
100	rectangular	cylinder	batch	Bubble	power	2	5000	0.09	85
225	rectangular	toroid	batch	Ep	linear	3	1000	0.01	40
400	rectangular	sheet	batch	Gaussian	inverse	1	3000	0.05	300
100	hexagonal	cylinder	batch	Ep	power	2	1000	0.01	17
225	hexagonal	toroid	batch	Gaussian	linear	3	3000	0.05	140
400	hexagonal	sheet	batch	Bubble	inverse	1	5000	0.09	450
100	hexagonal	cylinder	batch	Ep	linear	1	3000	0.09	50
225	hexagonal	toroid	batch	Gaussian	inverse	2	5000	0.01	230
400	hexagonal	sheet	batch	Bubble	power	3	1000	0.05	90
100	hexagonal	toroid	sequential	Ep	inverse	3	5000	0.05	1400
225	hexagonal	sheet	sequential	Gaussian	power	1	1000	0.09	630
400	hexagonal	cylinder	sequential	Bubble	linear	2	3000	0.01	7600
100	rectangular	sheet	batch	Ep	inverse	2	3000	0.05	49
225	rectangular	cylinder	batch	Gaussian	power	3	5000	0.09	236
400	rectangular	toroid	batch	Bubble	linear	1	1000	0.01	80

See Table 1 for the parameter definition.

The results of the numerical experiments associated with the design plan are used to find a linear regression model of the performance measure (E4) as function of the SOM's statistically significant parameters. The linear regression is performed in two stages: a stepwise regression [54] to select the statistically significant parameters and a linear regression fitting only on these to actually model the unknown mapping between them and the performance measure (E4). Stepwise regression parameters were: *Prob to Enter *= *0.05*, *Prob to Leave *= *0.05 *and *Direction *= *Mixed *and *Rules *= *No Rules*. The linear regression model is validated by comparing actual and predicted performance. JMP software was used for data analysis and linear regression fitting [55]. On the basis of this model the optimal values of the SOM parameters were derived.

#### Optimal sampling rate of MD trajectories

When dealing with a nanoseconds MD trajectory, it is desirable to train the SOM with a minimum number of selected structures while maintaining a reliable picture of the protein dynamics. Therefore, the effect of the sampling rate on the SOM learning process was assessed and the minimum number of frames to extract from a trajectory, without a statistically significant loss of information, was identified.

**Figure 2:**
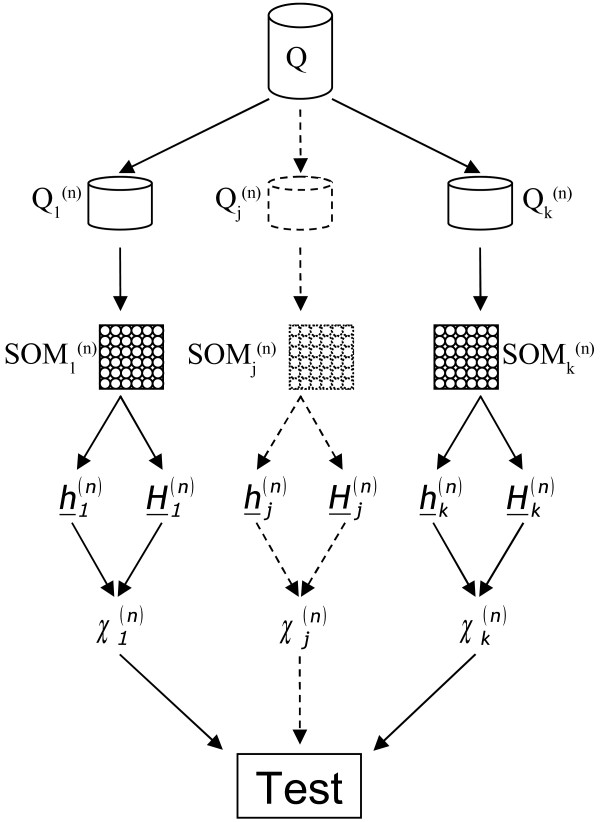
**Optimal sampling rate of MD trajectories**. Procedure for testing the SOM clustering at different sampling rates. The original data **Q **(40000 data points for each trajectory) is used to select the "optimal sampling rate of MD trajectories" between the following summarization levels; 20000; 10000; 5000; 2500; 1600; 800; 400; 80 and 40. For each summarization level "*n*", *k *samples Q_1_^(n)^,...,Q_k_^(n) ^were extracted. Each sample Q_i_^(n) ^summarizes a trajectory trough "*n*" randomly selected data points; each data point belongs to one of "*n*" equal width trajectory intervals. The "*k*" samples Q_1_^(n)^,...,Q_k_^(n) ^are used as learning data sets for SOM_1_^(n)^,...,SOM_k_^(n)^. Each SOM_i_^(n) ^is queried with data sets Q_i_^(n) ^and Q to obtain the hits pair (h_i_^(n)^, H_i_^(n)^) which is submitted to a Chi-squared goodness of fit test. In the case where the "*k*" goodness of fit tests are not rejected, the level of summarization "*n*" is assumed to summarize the original data set without a significant loss of information.

The rationale of the statistical procedure, applied to discover the "minimum number of frames" (Figure 2), is as follows: if the traini ng data set *Q^(n) ^*is representative of the original data set *Q*, then the SOM model *SOM^(n)^*, learnt by using the training data set *Q^(n)^*, when queried with data sets *Q^(n) ^*and *Q *must provide SOM hit distributions which are not statistically different.

In this study such a test was performed on three replicas by randomly selecting *n *conformations of the trajectory, each belonging to one of *n *equal width trajectory intervals. To better clarify the statistical procedure, let *Q_i_^(n) ^*be the *i^th ^*random sample extracted from *Q *and *SOM_i_^(n) ^*be the corresponding SOM trained on it by using the optimal parameters determined by experimental design. Furthermore, let *h_i_^(n) ^*and *H_i_^(n) ^*be the SOM hit vectors in the case where *Q_i_^(n) ^*and *Q *are presented to *SOM_i_^(n)^*. Then, the *χ_i_^(n) ^*Chi-square statistic is computed on non-null cells of the SOM hit vectors *H_i_^(n) ^*and *h_i_^(n) ^*while the null hypothesis is tested by the *n *Cressie-Read Chi-square test where the significance level has been set to α = 0.05 [56]. A diagram of the overall procedure is shown in Figure 2.

#### Cluster analysis of the SOM prototype vectors

The optimal SOM resulting from the previous optimization steps summarizes the conformational variability in the input data (structural ensembles generated by MD) through a set of prototype vectors. The next step of our approach consists on combining the prototype vectors (that can be interpreted as "protoclusters" [26]) in a reduced number of final clusters, where each original conformation belongs to the same cluster as its nearest prototype. To this end, two hierarchical agglomerative clustering algorithms, the *complete linkage *and the *average linkage*, were tested, following what described in [26].

Several clustering quality measures and stopping rules have been proposed to select the "optimal" number of clusters. Some of them were empirically shown to be more reliable [57] but there is no a universally accepted measure or rule. In this work the Mojena's stopping rule [58] was used for the following main reasons: it was placed in the set of the best performing stopping rules [57], especially when the number of clusters is not too big; it determines when a significant change from one stage to the next implies a partition which should not be undertaken; and it is computationally rather simple. However, other stopping rules and measures could have been fruitfully used among the first ten ranked in [57]. The Mojena's rule is simple and effective: the distances *d_i _*between the progressively merged clusters are recorded in order; from this set of distances, mean and standard deviation are calculated; the optimal number of clusters is equal to the largest *k *satisfying the following inequality:(E5)

where *sd_d _*is the standard deviation,  is the mean, and *z *is a specified constant.

A range of values for *z *were tested: 1.25, 1.50, 1.75, 2.00, 2.25, 2.50, and 2.75 for both complete and average linkage. Different values of the optimal number of clusters were obtained: from 5 to 8 for the complete linkage and from 4 to 10 for the average linkage. The most informative cluster separation was obtained with complete linkage and *z *= 2.75, and with average linkage and *z *= 2.50, which agrees with that suggested in [58].

The stability and better performance of the proposed two-level approach, i.e. SOM and linkage, with respect to other clustering methods, as well as the relative performances of the complete *vs*. average linkage algorithms were verified by the analysis reported in the Methods and Results sections on "Comparison of cluster analysis methods".

Hierarchical clustering was performed using the MATLAB Statistics Toolbox [59] and the Mojena's rule was applied in its MATLAB implementation, made available by courtesy of Prof. Fernández http://ima.udg.edu/~jamf/MARTIN_MF.htm.

### Comparison of cluster analysis methods

The proposed two-level approach for clustering structural ensembles (combining SOM with the average or the complete linkage methods) was compared with other clustering methods applied to the original input data vectors: GROMOS nearest neighbour [60], average and complete linkage methods [27]. GROMOS algorithm is implemented in the GROMACS package [31-33]: neighbours of each data point are defined according to a cutoff distance *c*; the point with largest neighbourhood defines the first cluster medoid; this point and its neighbours are removed and the algorithm is iterated until all data have been assigned to a cluster.

The quality of the clustering was assessed by the Silhouette (S) index [61] and the Davies-Bouldin (DB) index [62]. The two indices score the compactness and separations of the clusters, and were previously used in similar studies [5,26]. The S index can also be calculated per data point or cluster. Its value is in the range [-1, 1] and is generally interpreted as evidence in support of the cluster structure [63]: strong (S > 0.7), reasonable (0.5 < S ≤ 0.7), weak (0.25 < S ≤ 0.5) or no significant evidence (S ≤ 0.25). The DB is a measure of relative scattering of the data points. A lower value of DB indicates more compact and separated clusters.

The performance of the methods was compared for the number of clusters ranging from 2 to 15. In the case of GROMOS algorithm, solutions were generated for *c *ranging from 0.01 to 0.40 with increments of 0.005. Then the best solution for each number of clusters was selected according to the relevant quality index.

## Results

### Molecular Dynamics of the SH3 domain

**Figure 3:**
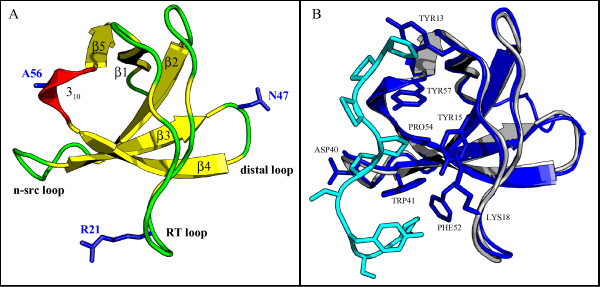
**Cartoon representations of the Spc-SH3 domain**. A) X-ray structure of WT Spc-SH3 (PDB code: 1SHG). Secondary structures are attributed according to the DSSP program (β-strands: yellow; 3_10 _helix: red; loops: green) and labelled according to the generally adopted nomenclature for SH3 domains. The three mutated residues are shown as blue sticks. B) Structure of the R21A Spc-SH3:p41 complex (PDB code: 2JMA), in blue, superimposed to the WT Spc-SH3 structure in grey. Residues that interact with the ligand are shown as sticks. The structure of p41 is shown in cyan.

The test case selected for our analysis is composed of the α-spectrin SH3 (Spc-SH3) domain and a group of its single-site mutants. The crystal structure of the wild-type domain (PDB code: 1SHG) [64] is characterized by two β-sheets, a short 3_10 _helix, and three connecting loops: the long RT loop that includes three isolated β-bridges, the n-src and the distal loop (Figure 3A). The Spc-SH3 domain binds the proline-rich decapeptide APSYSPPPPP (p41) [30]. The complex of the structurally similar R21A mutant with p41 [65] reproduces the typical features of the SH3 domains' binding [28,66], and the specificity pocket is formed by residues in the RT and n-src loops, and in the β4 strand (Figure 3B). Several studies demonstrated that the conformational dynamics of these loops plays an important role in defining the binding specificity of the SH3 domains [29].

The six mutants of the Spc-SH3 domain analysed here (and their PDB codes) are: R21A, R21G, A56G and A56S (2F2W, 2F2X, 2F2V and 2CDT) [30], N47A and N47G (1QKX, 1QKW) [67]. These mutants had been designed to explore how the local perturbations produced by single-point mutations affect both the stability and the p41 affinity of the domain [30].

In particular, it was observed that mutation of R21, at the tip of the RT loop that flanks the binding specificity pocket (Figure 3A), to alanine had favourable effects on the p41 affinity, probably due to the replacement of the bulky arginine side chain by a small side chain. On the contrary, a slight reduction in affinity was observed for the R21G mutant. Both mutations of N47, in the distal loop (Figure 3A), produced significant changes in the stability of the domain and a reduction of p41 affinity. Finally, while mutation of A56, in the 3_10 _helix belonging to one of the hydrophobic binding grooves (Figure 3A), to Ser did not alter significantly the p41 affinity, the mutation to Gly produced a great reduction in affinity.

The conformational dynamics of the wild type structure and the six mutated Spc-SH3 domains were analysed by 40 ns MD simulations (see the Methods section for details). Analysis of the root mean square distance (RMSD) to the starting structure confirmed a general stability of all trajectories, with an equilibration time around 1 ns (see Additional File 1) and a temperature around 300 K during the simulation. The covariance matrix overlap between the conformational spaces spanned by the two halves of the simulation and the overall trajectory for all the SH3 systems (Additional File 2) shows values in the range 0.70 - 0.87, confirming that the conformational subspaces explored in each half are representative of the complete space. Similar values were previously reported in cases of satisfactory sampling [15,39,68].

The most informative directions of motion were extracted by Essential Dynamics analysis. Analysis of the fraction of total motion described by different subspaces (Additional File 3) indicates that in all cases more than 79% is described by the first 20 eigendirections (corresponding to 12% of the total space), while the first 30 directions (18% of total space) can explain more than 85% (in most cases more than 90%) of the conformational flexibility of all domains. Therefore, the 30 dimensional essential space of each system was selected for the following analyses. In this way, the noise given by not informative high-frequency motion was removed.

The choice of projecting the trajectories in the essential space led to an increased signal to noise ratio in the plots of the RMSF on the positions of the Cα atoms (E2). A set of comparative RMSF plots is shown in Additional File 4: the WT SH3 structure is rather constrained, with only a region of flexibility in the n-src and the distal loops (Additional File 4a), while the effects of single-point mutations in each of the three positions, R21, N47 and A56 (see Figure 3A), can be clearly observed. While R21A mutation does not affect the overall domain flexibility, the R21G mutation causes a relevant increase of flexibility in the long RT loop and in the region including β3, the distal loop, β4 and the helix 3_10 _(Additional File 4b). These differences suggest that flexibility may have a role in the different binding affinities of the two mutants, that could not be explained only on the basis of the very similar stereo-electronic characteristics of their binding sites [30]. The mutation to Ala at position 47 has little effect on the flexibility, while a change to Gly in the same position causes an increased flexibility in the surrounding region as well as in the whole RT loop (Additional File 4c). This confirms the hypothesis of a long-range propagation of the local perturbation to the binding site, that may have a role in the N47G binding to p41 [69]. Different from previous cases, a mutation to Gly in position 56, in the 3_10 _helix, does not alter either the local or the global flexibility of the domain; an increase of flexibility in the distal loop region is observed for the A56S mutant (Additional File 4d). From these results, the conformational freedom of these two mutants does not seem to be related to the p41 binding affinity [30].

### SOM optimization to analyse the SH3 domain dynamics

Following the protocol described in the Methods section, a SOM was optimized for the analysis of the MD trajectories of the WT SH3 and its six mutants. Each sampled conformation was described by the Cartesian coordinates of the Cα atoms projected on the Essential Space. The 55 Cα atoms corresponding to structurally equivalent positions in all the domains were included in the analysis; therefore, the input data presented to the SOM were vectors of 165 elements.

The optimal values for the SOM parameters were found by experimental design using a Taguchi design plan (see Table 2) which consists of 36 runs. Each run was replicated three times on four data sets:

○ WT

○ R21G

○ WT and R21G

○ ALL (WT and the six mutants),

thus resulting in a total of 432 experiments. The choice of multiple and combined data sets aimed at optimizing the map both for the analysis of a single trajectory and for multiple comparisons of different trajectories.

The response variable was the minimum normalized distance defined in equation (E4). The summary of the linear regression model and the significance analysis of the single SOM parameters are reported in Table 3 and 4: the regression model is satisfactory, with similar values of R^2 ^and R^2^_adj _(R^2 ^= 0.937 and R^2^_adj _= 0.936). The following SOM parameters have been judged to be statistically significant at the p-value cut-off of 0.01: *Map size*, *Radius*, *Training length, Neighbour function *and *TYPEMOL *(Table 4), the latter being a categorical variable, which can take the following values: WT, R21G, WT+R21G and ALL. It is worthwhile to mention that such parameters are statistically significant for all the values of the categorical variable *TYPEMOL*. Furthermore, the optimal setting of the SOM parameters, for each value of the categorical variable *TYPEMOL*, is the same: *Map size *= 100, *Radius *= 3, *Training length *= 5000 and *Neighbour function *= gaussian. Table 5 reports the results of a validation test on the regression model (see Methods for details). The difference between the predicted and the actual optimal values is small, confirming that the regression model can reliably predict the value of the performance measure (E4) as a function of the statistically significant SOM parameters.

**Table 3:** Linear regression, summary of fit.

Parameter	Value
R^2^	0.937
R^2^_adj_	0.936
Mean of Response	395.01
RMSE (Root Mean Square Error)	27.61

Observations	432

**Table 4:** Linear regression, effect tests [55].

Source	Nparm	DF	Sum of Squares	F Ratio	Prob > F
Map size	1	1	1022810.9	1341.853	<0.0001
Radius	1	1	39779.1	52.187	<0.0001
Training length	1	1	17056.8	22.377	<0.0001
Neighbour function	2	2	2935552.0	1925.615	<0.0001
TYPEMOL	3	3	805604.4	352.298	<0.0001

See Table 1 for the parameter definition.

**Table 5:** Optimal SOM parameter values.

Case	Map size	Radius	Training length	Neighbour function	Actual	Predicted	Time (s)
WT	100	3	5000	gaussian	158	156	50
R21G	100	3	5000	gaussian	265	270	50
WT+R21G	100	3	5000	gaussian	259	238	105
ALL	100	3	5000	gaussian	254	246	340

See Table 1 for the parameter definition.

The SOM optimization was performed on conformations sampled with a rate of 1/100 ps, randomly selecting a structure in each interval of 100 ps to avoid a correlation bias. This initial choice was validated and confirmed *a posteriori *in a test on the impact of different sampling rates (see Methods section for details). The analysis was performed with three replicas for each sampling rate: 1/2 ps, 1/4 ps, 1/8 ps, 1/16 ps, 1/25 ps, 1/50 ps, 1/100 ps, 1/500 ps and 1/1000 ps (corresponding to sampling size from 20000 to 40 conformations): 20000, 10000, 5000, 2500, 1600, 800, 400, 80, and 40 conformations. The results for a test set composed by WT, R21G and N47A, are reported in Table 6 showing that the null hypothesis is not rejected for samples larger than 400 conformations. Thus, at a rate of 1/100 ps or greater the results of the SOM analysis are not influenced by the sampling rate.

**Table 6:** Test on the effects of different sampling rates for the WT SH3 and the R21G and N47A mutants.

		statistic			p-value			result		
**number of conformations**	**sample**	**WT**	**R21G**	**N47A**	**WT**	**R21G**	**N47A**	**WT**	**R21G**	**N47A**

20000	1	25	27	16	1.00	1.00	1.00	-	-	-
	2	17	28	17	1.00	1.00	1.00	-	-	-
	3	19	29	22	1.00	1.00	1.00	-	-	-

10000	1	29	35	34	1.00	1.00	0.98	-	-	-
	2	28	37	36	1.00	1.00	0.98	-	-	-
	3	33	36	21	0.99	1.00	1.00	-	-	-

5000	1	30	33	30	1.00	1.00	0.98	-	-	-
	2	49	49	28	0.91	0.98	0.98	-	-	-
	3	34	37	30	0.99	1.00	0.99	-	-	-

2500	1	63	53	23	0.26	0.92	0.99	-	-	-
	2	51	48	56	0.76	0.99	0.22	-	-	-
	3	39	44	34	0.92	0.99	0.98	-	-	-

1600	1	43	49	31	0.82	0.93	0.96	-	-	-
	2	42	63	28	0.86	0.74	0.95	-	-	-
	3	45	48	41	0.90	0.97	0.77	-	-	-

800	1	40	57	73	0.92	0.89	0.06	-	-	-
	2	57	43	49	0.52	0.99	0.47	-	-	-
	3	54	44	36	0.48	0.98	0.80	-	-	-

400	1	48	73	39	0.63	0.11	0.49	-	-	-
	2	50	49	55	0.55	0.93	0.16	-	-	-
	3	34	39	46	0.96	0.99	0.47	-	-	-

80	1	40	83	54	0.28	0.00	0.02	-	*	*
	2	63	64	38	0.00	0.00	0.08	*	*	-
	3	81	46	50	0.00	0.43	0.04	*	-	*

40	1	167	84	67	0.00	0.00	0.00	*	*	*
	2	105	62	95	0.00	0.00	0.00	*	*	*
	3	101	60	67	0.00	0.00	0.00	*	*	*

*k *= 3 and *α *= 0.05. Statistic, p-value and the output of the hypothesis test are reported; (-) means that the null hypothesis cannot be rejected while (*) means that the null hypothesis has been rejected.

### Comparison of cluster analysis methods

**Figure 4:**
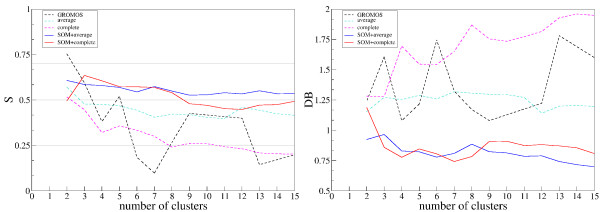
**Comparison of cluster analysis methods**. The performance of different methods on the ALL data set was assessed by the Silhouette (left) and the Davies-Bouldin (right) indices.

The two-level approach was compared with three cluster analysis methods: GROMOS [60], average linkage and complete linkage. The tests were performed on the ALL (WT and the six mutants) data set and assessed by the Silhouette (S) [61] and Davies-Bouldin (DB) indices [62] (see Methods section for details). The results are reported in Figure 4.

The two-level approach proposed in this work (SOM and linkage) is the best performing: the S (DB) scores are generally higher (lower) for all the number of clusters and the profiles are more stable. This suggests that the SOM preclustering is reducing the noise and generating more compact and well separated clusters. This is also confirmed by the per-cluster values of S (see Additional file 5). According to S, only the two-level approach shows reasonable evidence of correct cluster structure (S > 0.5) for most values of the number of clusters range.

While from the analysis of the two global S and DB indices both the linkage methods appear to be good choices for our two-level approach, a more detailed analysis of the performances on the ALL dataset revealed some differences. In particular, the complete linkage provides clearer and better separated clusters for conformations related to the most characteristic motions of the domain exploited by specific mutants (see the discussion of Figure 7 in the next section). Accordingly, the complete linkage algorithm was used in association with SOM for conformational and functional analysis of the SH3 domains.

The S and DB indices are often employed to identify the optimal number of clusters. In this case they show a rather even profile for the two-level approach, but it has to be noted that the significant values for the SOM and complete linkage approach range from 3 to 8. This is compatible with the optimal value of 5 that was selected by the Mojena's rule for the ALL dataset.

Additional File 6 shows a 2D RMSD matrix of the conformations in the ALL data set, where the lower triangular part reports the best 5 cluster solutions with GROMOS. While the RMSD maps are powerful exploratory tools for single trajectories, they become increasingly complex when several simulations are reported. Additionally the image shows a limitation of GROMOS nearest neighbour approach: a tendency to generate singletons. This is also a frequent event in single linkage clustering [5] that for this reason was not included in the test.

### Conformational and functional analysis of the SH3 domains by SOM and complete linkage clustering

The proposed approach (SOM and complete linkage) with the SOM parameters and the sampling rate values set in the optimization stage was used to perform a conformational analysis of the MD trajectories of single SH3 domains independently as well as groups of simulations to detect similarities and differences in flexibilities among the systems. We will present the results for the following ensembles: R21G, WT and R21G, WT and six mutants.

**Figure 5:**
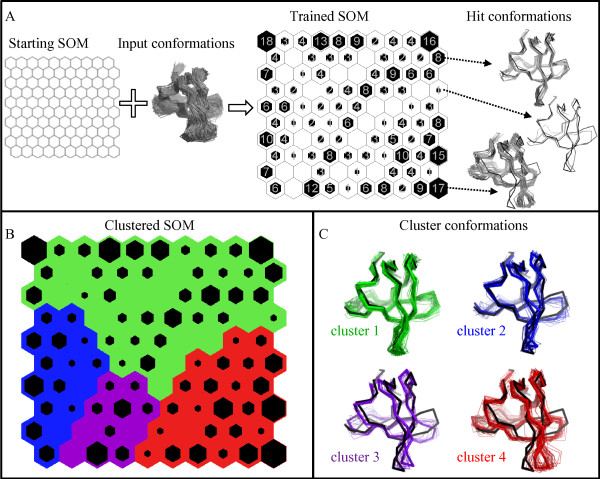
**SOM analysis of the R21G mutant dynamics**. A) Training phase of the SOM. From left to right: " Starting SOM" composed by the empty hexagons (neurons) before the training process; "Trained SOM" reporting the number of hits in each neuron both as grey numbers and with black hexagons proportionally sized; "Hit conformations" for three neurons (taken as examples) represented as grey tubes (*neuron level*). B) "Clustered SOM" (see Methods section for a detailed description): the four clusters obtained by hierarchical clustering of the neurons are indicated by different colors. C) Tube representation of the ensemble of the hit conformations closest to the neuron prototype vectors in each cluster (*cluster level*); the hit conformation closest to the cluster centroid (*centroid level*) is highlighted by a larger tube. In all the conformational ensembles the X-ray structure of the WT SH3 is reported in black for comparison.

The map of the R21G ensemble is shown in Figure 5 as an example of analysis of a single trajectory. As described in the Methods section, first, the SOM learning process produces neuron prototype vectors that are made similar to the input conformations "won" during this phase (hit conformations), second, prototype vectors are subjected to hierarchical clustering. Therefore, it is possible to extract information at three different levels: *neuron level*, *i.e*. the ensemble of hit conformations in a neuron; *cluster level*, *i.e*. the ensemble of hit conformations closest to the prototype vectors in a cluster; *centroid level*, *i.e*. the hit conformation closest to the cluster centroid. Four clusters (Figure 5B) were extracted applying the Mojena's rule after complete linkage clustering and they are represented at both the cluster and the centroid levels in Figure 5C. All the extracted conformations are superimposed to the X-ray structure of the WT SH3 (in black) taken as reference. Cluster 1 (green) contains a large group of representative conformations with limited fluctuations mostly localised in the n-src loop. Clusters 2 (blue) describes a small displacement of the distal loop towards the RT loop, while cluster 3 (violet) comprises conformations with a more extended motion of the same loop, a consequent perturbation of the n-src loop, and a motion of the RT loop tip back towards the distal loop. Conformations in cluster 4 (red) greatly deviate from the WT structure, with a concerted closure motion of the distal and RT loops.

**Figure 6:**
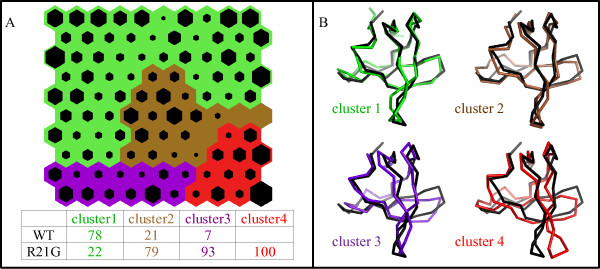
**SOM analysis for the WT SH3 and the R21G mutant dynamics**. A) Self-Organising Map (see Figure 5 and Methods section for a detailed description); the percentage distribution of conformations of each domain in the clusters is reported in the table. B) Tube representation of the hit conformation closest to each cluster centroid (centroid level), superimposed to the X-ray structure of the WT SH3 (in black).

Four clusters of neurons were extracted from the map trained on the WT and R21G sets of conformations. A table of cluster compositions is reported, along with the map, in Figure 6A and the representative conformation of each cluster (centroid level) is shown in Figure 6B. The WT ensemble, characterized by low fluctuations, contributes to 78% of cluster 1, that includes conformations similar to its equilibrium structure, and part of cluster 2, whose conformations present small fluctuations in the distal and n-src loops. On the contrary, cluster 3 and 4 are almost exclusively populated by conformations from the R21G trajectory. Cluster 3 describes more extended fluctuations in the distal and n-src loops and in part of the RT loop, and cluster 4 large concerted motions in the distal and the faced RT loop.

**Figure 7:**
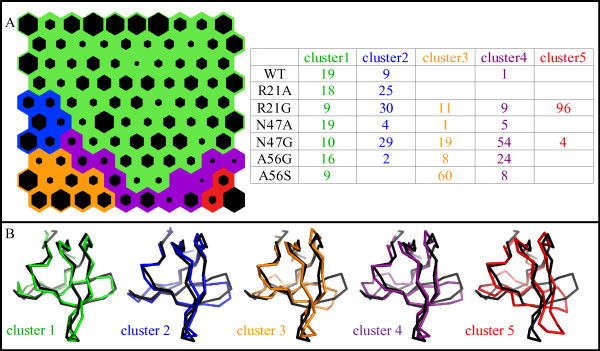
**SOM analysis of the dynamics of the WT SH3 and the six mutants**. A) Self-Organising Map (see Figure 5 and Methods section for a detailed description); the percentage distribution of conformations of each domain in the clusters is reported in the table. B) Tube representation of the hit conformation closest to each cluster centroid (centroid level), superimposed to the X-ray structure of the WT SH3 (in black).

The SOM obtained through learning from the whole set of ensembles and the resulting five clusters are shown in Figure 7 at the centroid level. The representative conformations of cluster 1 and 5 (Figure 7B) closely resemble those of cluster 1 (low fluctuations) and 4 (large concerted motion of the distal and RT loops) also observed in the SOM of WT and R21G (Figure 6B). Clusters 2, 3 and 4 describe intermediate situations. While cluster 2 describes a moderate flexibility of the distal loop and cluster 3 a large flexibility of the same loop associated with perturbation of the n-src and the RT loops, cluster 4 describes a perturbation distributed to the whole domain. For cluster 4, the presence of low populated or empty neurons in the neighbourhood of cluster 5 highlights its role in effectively separating clusters 2 and 3 from cluster 5. The inspection of the per-cluster values of the Silhouette index (Additional file 5) confirms this role: while strong cluster structure is evidenced for clusters 2, 3 and 5 (0.70 ≤ S ≤ 0.95), a very low value (S = 0.11) is associated to cluster 4 (see Methods section). The ability of the complete linkage algorithm to clearly separate clusters describing the most characteristic motions of the domain (clusters 2, 3 and 5) confirmed the choice of using this method in association with SOMs.

**Figure 8:**
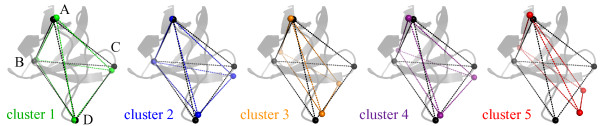
**Distances among four selected points in each cluster**. Dotted lines are coloured according to clustering reported in Figure 7. The same distances in the WT SH3 structure are reported in black dotted lines. The X-ray structure of the WT SH3, taken as a reference, is represented by grey cartoons and superimposed onto each graph. Points A, B, C, D, are the Cα atom positions in the representative conformation of each cluster of the following residues: A = L12; B = S36 in the n-src loop; C = D48 in the distal loop; D = P20 at the tip of the RT loop.

To complement and confirm this analysis, the dRMSD (E3) between the distances of four selected points in the average conformation of each cluster and in the X-ray structure of the WT SH3 was calculated. The selected points (see Figure 8) are the Cα atoms of a constrained residue at the N-term of the RT loop (A = L12), and three residues in the most flexible regions of the protein (B = S36 in the n-src loop, C = D48 in the distal loop, D = P20 at the tip of the RT loop). Both the visual analysis of the inter-point distances in Figure 8 and the ABCD dRMSD values (Table 7) indicate that clusters 1 and 2 slightly deviate from the WT structure, where clusters 3 and 4 have more relevant deformations (around 2 Å) and cluster 5 departs more than 3 Å from the WT structure. In detail deformations in the average structure of cluster 3 mainly affect the distal and n-src loops' distances (ABC), while in clusters 4 and 5 the distances of the RT loop from both the other loops and the reference point A (ACD and ABD) depart from the WT geometry.

**Table 7:** dRMSD values between points A, B, C, D, in Figure 8.

	dRMSD (Å)			
	ABD	ACD	ABC	ABCD
Cluster 1	1.7	0.9	1.4	1.4
Cluster 2	1.0	1.5	1.6	1.4
Cluster 3	2.2	2.3	2.7	2.4
Cluster 4	2.0	2.1	1.6	2.2
Cluster 5	3.8	2.9	2.4	3.5

The dRMSD are calculated as the average distance of the selected points in the conformational ensemble of each cluster and the same distance in the WT SH3 structure.

A closer look at the cluster composition shows the ability of the SOM to group conformations common to all domains, as well as to correctly separate the typical dynamics of each of the three domains with higher flexibility. The contributions of each mutant ensemble to the five clusters (Figure 7A) highlights that clusters 1 and 2 are populated by conformations from all the mutants. The larger contributions to cluster 1 are from the WT SH3 and the mutants with reduced conformational flexibility (R21A, N47A and A56G), while cluster 2 is more representative of R21G and N47G ensembles. Each of the remaining three clusters is dominated by one contribution: cluster 3 mainly by A56S, cluster 4 by N47G and cluster 5 is almost completely populated by conformations from the R21G ensemble.

An interesting feature arises from the topological nature of the SOM. Conformational transitions that occur in consecutive times along the MD trajectory involve conformations assigned to neighbour clusters on the map. An example for three trajectories is reported in Additional File 7 where the obtained clusters are annotated on the RMSD plots by colours. In the first part of the WT RMSD plot, frequent transitions occur between conformations in the green and blue clusters that are neighbours in the SOM. More clearly, in the A56S plot, transitions between conformations in the green and yellow clusters (that in the SOM are separated by the violet cluster) always occur in the trajectory through sampling of conformations in the violet cluster. In the R21G plot, while some transitions occur between conformations in neighbour clusters (blue and green), others (green to yellow or red to yellow) are separated by the violet cluster in the map and occur only through brief sampling of conformations of this type.

Previous studies suggested a hypothesis of the role of conformational flexibility in reducing the binding affinity [30]. To verify this we studied the effects of flexibility on the binding site geometry. As previously described, the binding pocket of the p41 peptide is flanked by the RT and n-src loops (Figure 3B), whose dynamics affects the binding specificity. Therefore, the inter-residue distances in the binding site of the SH3:p41 complex (all the heavy atoms in the residues interacting with p41, shown in Figure 3B) in the representative conformation of each cluster were compared to those in the experimental structure. The dRMSD values confirmed that the increase of conformational freedom induced by the N47G and R21G mutations (mainly described by clusters 4 and 5) produces a significant distortion of the binding site geometry (dRMSD = 1.7 and 1.8 Å), while other mutations did not produce comparable effects (dRMSD around 1 Å). These results correlate well with the binding affinity data reported for the N47G and R21G mutants [30]. In fact, even if the site of the former mutation is distant from the binding site, a significant decrease in binding affinity is produced. Moreover the R21G mutant that, on the basis of the favorable changes in the binding site stereo-electronic properties, was expected to give a significant increase in binding affinity to p41 (as observed for the R21A mutant), indeed produced a slight reduction in affinity.

## Discussion

We have presented a strategy to compare conformational ensembles of protein domains with the goal of highlighting similarities and differences in functional motions. This strategy uses SOMs, that have recently been shown to be suitable for the analysis of individual MD trajectories [5], but takes also advantage of a two-level approach [26] where complete linkage clustering is performed on the SOM prototype vectors. To provide the users with optimal SOM parameters for this specific type of data, we developed a protocol by which we identified a small number of important parameters and calculated their optimal values (Table 5).

When dealing with large data sets of conformations, a major issue is the computational cost of the analysis. A possible strategy to overcome this problem is to compare only the average geometrical properties of subgroups of data, for example the ensemble RMSF on atom positions. However in this analysis small fluctuations are difficult to detect, the direction of motion associated to each peak is not considered, and comparisons are only pair-wise. These limitations appeared clearly in our analysis of the RMSF profiles of the Spc-SH3 domains. On the contrary, the proposed SOM approach retains high sensitivity, is able to differentiate motions with similar average fluctuation and is not restricted to pair-wise comparisons. A second strategy to reduce computational costs involves using geometrical clustering methods in a two-stage or sieved approach [5] by initially clustering only part of the data and then in a second step by adding the missing ones into existing clusters. However, this can lead to the loss or distortion of the topological relations among the original data and eventually to a biased grouping, if the selection at the first stage is not representative. Similarly during the SOM training, each data vector is compared only to the neuron vectors representing all the data already presented to the map, but the topological relations are intrinsically recorded and the representative geometries are dynamically updated avoiding a bias. This comes at increased computational cost in the training stage, but once a map is trained on a group of representative protein domains, it can be used for fast classification of conformational ensembles of similar systems.

In the proposed two-level approach, where complete linkage is applied on the SOM prototype vectors, the number of neurons identified by experimental design (*Map size *= 100) allows the SOM model to optimally capture the relevant features of a large input space in a smaller set of prototype vectors. These can be interpreted as an intermediate topological reference ("protoclusters" [26]), which allows efficient use of clustering algorithms to divide the prototypes into groups. The number of clusters has not to be specified in advance but it is identified only after hierarchical clustering on the prototype vectors by the Mojena's stopping rule. This generates a significant reduction in computational time and in noise compared to the direct application of linkage algorithms to the input data [26]. This also differs significantly from the approach adopted in a previous study by other authors [5] where only a SOM was used for clustering the original data and the number of clusters had to be selected in advance by the user, *i.e*. before the SOM learning takes place.

The results for the SH3 domains highlighted the specific advantages of a SOM in conformational and functional analysis. The major benefit is the possibility of providing a topological mapping of the conformational space embedded in a simple 2D visualisation that simplifies the identification of differences in the conformational dynamics (see Figures 5, 6, 7). Moreover, the map can adapt to record differences in both large and small fluctuations, as well as to group conformations associated with different directions of the same motion.

The proposed approach resulted in a very efficient comparison of multiple trajectories: low fluctuations, large concerted motions and intermediate dynamic perturbations were clearly and correctly detected (Figures 7 and 8, and Table 7). The comparison of inter-residue distances in the binding site [65] among the cluster representative conformations led to a functional interpretation of the observed differences. The increase of conformational freedom induced by the N47G and R21G mutations induces a distortion of the binding site geometry that explains the decreased ligand binding ability, while other mutations do not produce comparable effects.

The comparison of the two-level approach to other clustering methods showed that adding a SOM preclustering stage has the benefit of reducing the noise and generating more compact and well separated clusters (see Figure 4 and Additional file 5).

Our approach can be reliably used on other study-cases. Indeed, the number of statistically significant SOM parameters is small while their optimal values are expected to be suitable for conformational spaces with similar complexity, *i.e*. similar number of unique conformations sampled, to the SH3 study case. In particular, the optimal values of the three parameters associated with the SOM training process (*Radius *= 3, *Training length *= 5000 and *Neighbour function *= Gaussian) are expected to generalize quite well. The *Map size *parameter is more critical; the optimal value we found (100) allows the summarization of a great number of unique features, but it could be necessary to re-set it specifically for cases with a greater variance in the training data.

The approach presented here is independent from the method used to generate the structural ensemble and is reliable to describe both small and large differences. It is therefore suitable to also analyse combinations of ensembles from computational methods with a more extended sampling of the conformational space and from experiments (NMR ensembles or multiple X-ray depositions of the same structure).

The test-case presented here includes mutants of a single domain that can easily be aligned. A future development of this study is the identification of alternative representations of protein conformations that do not require the preliminary definition of structurally equivalent positions by structural alignment. This will allow an extension to the comparison of different domains, including distant homologous proteins.

## Conclusions

The novel approach presented here to analyse conformational ensembles of protein domains resulted in a very efficient way of comparing multiple trajectories of the test case and analysing the role of conformational flexibility in modulating the domain function. The approach can easily be extended to other study cases. Further applications may include the treatment of ensemble of conformations derived from other sources of dynamical data, and the use of a trained map to classify conformational ensembles of similar systems. Future directions involve the extension of the approach to the comparison of protein flexibilities also for distant homologous proteins.

## Authors' contributions

DF, AP and LB jointly conceived the study. DF performed all calculations and data analysis. FS participated in the design and set up of the SOM protocol. AP and LB participated in the Molecular Dynamics studies and in the data analysis. DF, AP, FS and LB wrote the manuscript.

## Supplementary Material

Additional file 1 **Plot of RMSD to the starting structure during the MD simulations**. The plots report, for all the systems, the RMSD values in the whole MD trajectory and a snapshot of the first 2 ns, to highlight the equilibration time. Click here for file

Additional file 2 **Overlap of sampling in the MD simulations of the SH3 domains**. The values represent the overlap (E1) between the conformational spaces spanned by each half of the simulation and that of the overall trajectory. Click here for file

Additional file 3 **Distribution of motion in different subspaces for each MD simulation**. Values refer to the percentage of total space described by the eigenvectors. Click here for file

Additional file 4 **Plot of RMSF versus residue position in the essential space**. MD simulations of: a) WT SH3; b) R21A and R21G mutants, compared to the WT; c) N47G and N47A mutants, compared to the WT; d) A56G and A56S mutants, compared to the WT. Only equivalent residues in the preliminary structure-based alignment are included and their numbering is modified according to the alignment. Secondary structures are reported in the bottom part of each graph for reference; they are attributed according to the DSSP program (β-strands: black squares; 3_10 _helix: white square) and labelled following the nomenclature generally adopted for SH3 domains. Click here for file

Additional file 5 **Comparative Silhouette plots**. The panels report the cluster quality for average linkage, complete linkage and the corresponding two-level approach (SOM and average linkage or SOM and complete linkage). In each panel Silhouette profiles are plotted for the number of clusters ranging from 2 to 10. A Silhouette profile is composed by a bar for each identified cluster. Click here for file

Additional file 6 **Distance matrix and GROMOS clustering**. The distance (RMSD) matrix for the combined trajectories of the ALL data set is reported in the upper triangular part of the image. The cluster attribution generated by GROMOS for a number of clusters equal to five is reported in the lower triangular part. Click here for file

Additional file 7 **Plot of RMSD versus time during the MD simulations**. From top to bottom, MD trajectories for the WT SH3, A56S and R21G mutants. Conformations attributed to the five clusters obtained from the SOM trained on the entire group of trajectories are coloured according to Figure 7. Click here for file
