# Supplementary material for: Conformational and functional analysis of molecular dynamics trajectories by Self-Organising Maps
Source: BMC Bioinformatics. 2011 May 14;12:158. doi: 10.1186/1471-2105-12-158 (PMC3118354; doi:10.1186/1471-2105-12-158)

Plot of RMSD to the starting structure during the MD simulations

The plots report, for all the systems, the RMSD values in the whole MD trajectory

and a snapshot of the first 2 ns, to highlight the equilibration time

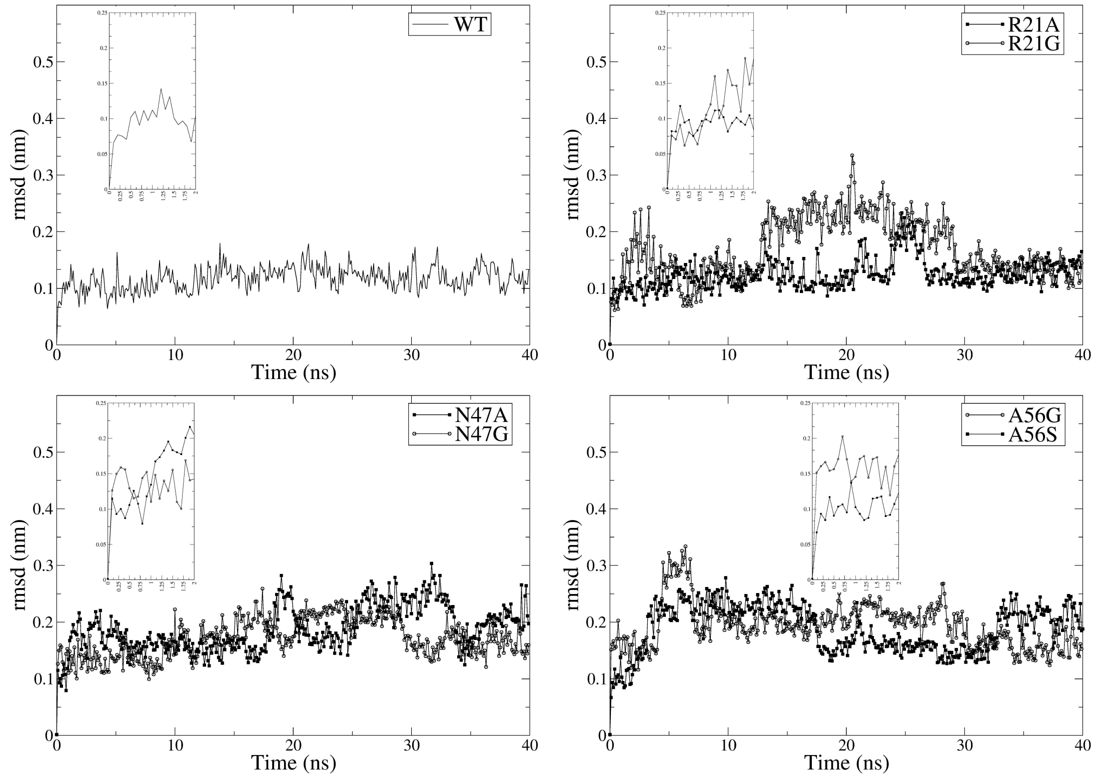

Supplement: Additional file 1 — Plot of RMSD to the starting structure during the MD simulations. The plots report, for all the systems, the RMSD values in the whole MD trajectory and a snapshot of the first 2 ns, to highlight the equilibration time. [file 1471-2105-12-158-S1.PDF]
