# Supplementary material for: Conformational and functional analysis of molecular dynamics trajectories by Self-Organising Maps
Source: BMC Bioinformatics. 2011 May 14;12:158. doi: 10.1186/1471-2105-12-158 (PMC3118354; doi:10.1186/1471-2105-12-158)
Supplement: Additional file 2 — Overlap of sampling in the MD simulations of the SH3 domains. The values represent the overlap (E1) between the conformational spaces spanned by each half of the simulation and that of the overall trajectory. [file 1471-2105-12-158-S2.PDF]

Overlap of sampling in the MD simulations of the SH3 domains.

The values represent the overlap (E1) between the conformational spaces spanned by each half of the simulation and that of the overall trajectory.

	1 - 20 ns	20 - 40 ns
WT	0.87	0.86
R21A	0.76	0.77
R21G	0.82	0.78
N47A	0.70	0.75
N47G	0.72	0.78
A56G	0.71	0.71
A56S	0.83	0.80
