# Supplementary material for: Conformational and functional analysis of molecular dynamics trajectories by Self-Organising Maps
Source: BMC Bioinformatics. 2011 May 14;12:158. doi: 10.1186/1471-2105-12-158 (PMC3118354; doi:10.1186/1471-2105-12-158)
Supplement: Additional file 3 — Distribution of motion in different subspaces for each MD simulation. Values refer to the percentage of total space described by the eigenvectors. [file 1471-2105-12-158-S3.PDF]

Distribution of motion in different subspaces for each MD simulation

Values refer to the percentage of total space described by the eigenvectors.

Eigenvectors	WT	R21A	R21G	N47A	N47G	A56G	A56S
1 - 10	65.2	69.9	81.5	73.5	77.1	80.0	80.3
1 - 20	79.3	82.1	89.4	85.2	87.0	88.8	88.6
1 - 30	86.3	88.0	92.8	90.4	91.4	92.5	92.3
1 - 60	95.0	95.5	97.3	96.5	96.7	97.1	97.1
1 - 165	100.0	100.0	100.0	100.0	100.0	100.0	100.0
