# Supplementary material for: Conformational and functional analysis of molecular dynamics trajectories by Self-Organising Maps
Source: BMC Bioinformatics. 2011 May 14;12:158. doi: 10.1186/1471-2105-12-158 (PMC3118354; doi:10.1186/1471-2105-12-158)

Plot of RMSF versus residue position in the essential space.

MD simulations of: a) WT SH3; b) R21A and R21G mutants, compared to the WT; c) N47G and N47A mutants, compared to the WT; d) A56G and A56S mutants, compared to the WT. Only equivalent residues in the preliminary structure-based alignment are included and their numbering is modified according to the alignment. Secondary structures are reported in the bottom part of each graph for reference; they are attributed according to the DSSP program (β-strands: black squares; 3_{10} helix: white square) and labelled following the nomenclature generally adopted for SH3 domains

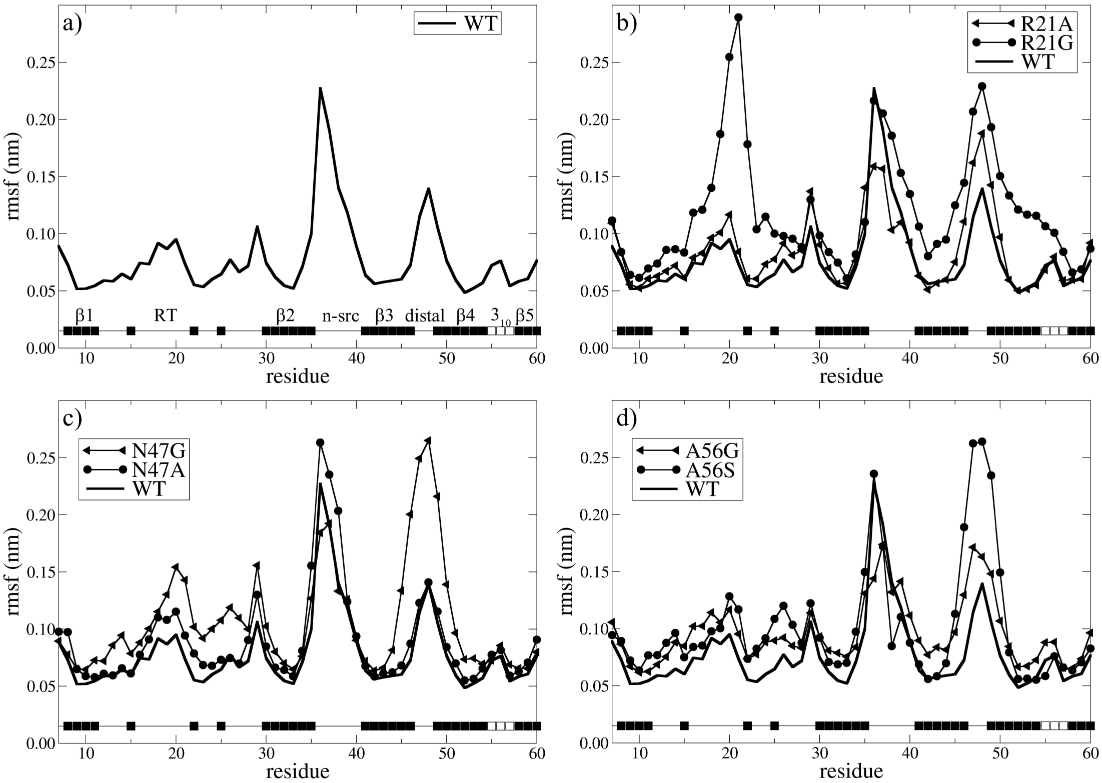

Supplement: Additional file 4 — Plot of RMSF versus residue position in the essential space. MD simulations of: a) WT SH3; b) R21A and R21G mutants, compared to the WT; c) N47G and N47A mutants, compared to the WT; d) A56G and A56S mutants, compared to the WT. Only equivalent residues in the preliminary structure-based alignment are included and their numbering is modified according to the alignment. Secondary structures are reported in the bottom part of each graph for reference; they are attributed according to the DSSP program (β-strands: black squares; 310 helix: white square) and labelled following the nomenclature generally adopted for SH3 domains. [file 1471-2105-12-158-S4.PDF]
