# Supplementary material for: Conformational and functional analysis of molecular dynamics trajectories by Self-Organising Maps
Source: BMC Bioinformatics. 2011 May 14;12:158. doi: 10.1186/1471-2105-12-158 (PMC3118354; doi:10.1186/1471-2105-12-158)

Comparative Silhouette plots

The panels report the cluster quality for average linkage, complete linkage and the corresponding two-level approach (SOM and average linkage or SOM and complete linkage). In each panel Silhouette profiles are plotted for the number of clusters ranging from 2 to 10. A Silhouette profile is composed by a bar for each identified cluster.

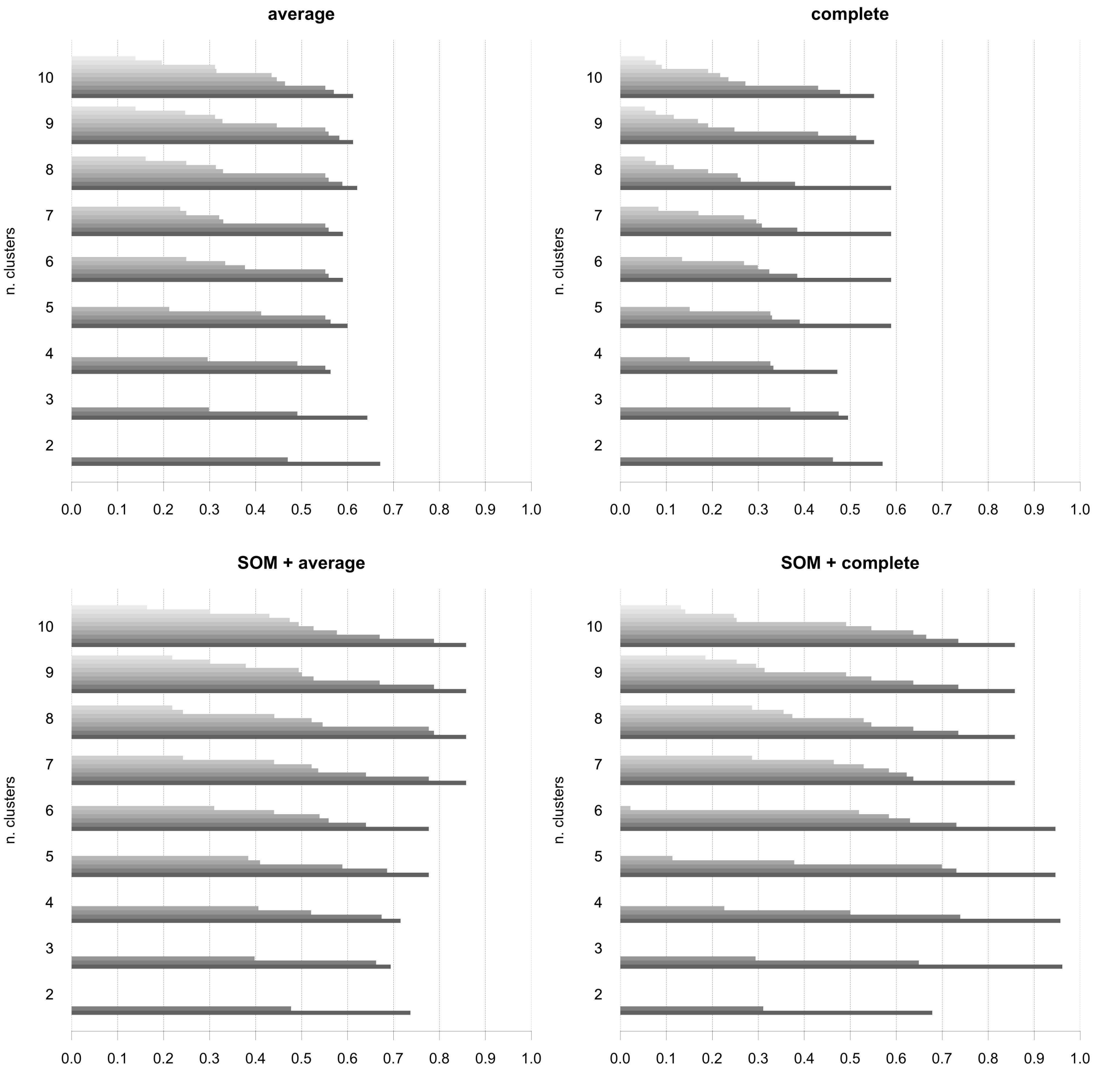

Supplement: Additional file 5 — Comparative Silhouette plots. The panels report the cluster quality for average linkage, complete linkage and the corresponding two-level approach (SOM and average linkage or SOM and complete linkage). In each panel Silhouette profiles are plotted for the number of clusters ranging from 2 to 10. A Silhouette profile is composed by a bar for each identified cluster. [file 1471-2105-12-158-S5.PDF]
