# Supplementary material for: Conformational and functional analysis of molecular dynamics trajectories by Self-Organising Maps
Source: BMC Bioinformatics. 2011 May 14;12:158. doi: 10.1186/1471-2105-12-158 (PMC3118354; doi:10.1186/1471-2105-12-158)

Distance matrix and GROMOS clustering.

The distance (RMSD) matrix for the combined trajectories of the ALL data set is reported in the upper triangular part of the image. The cluster attribution generated by GROMOS for a number of clusters equal to five is reported in the lower triangular part.

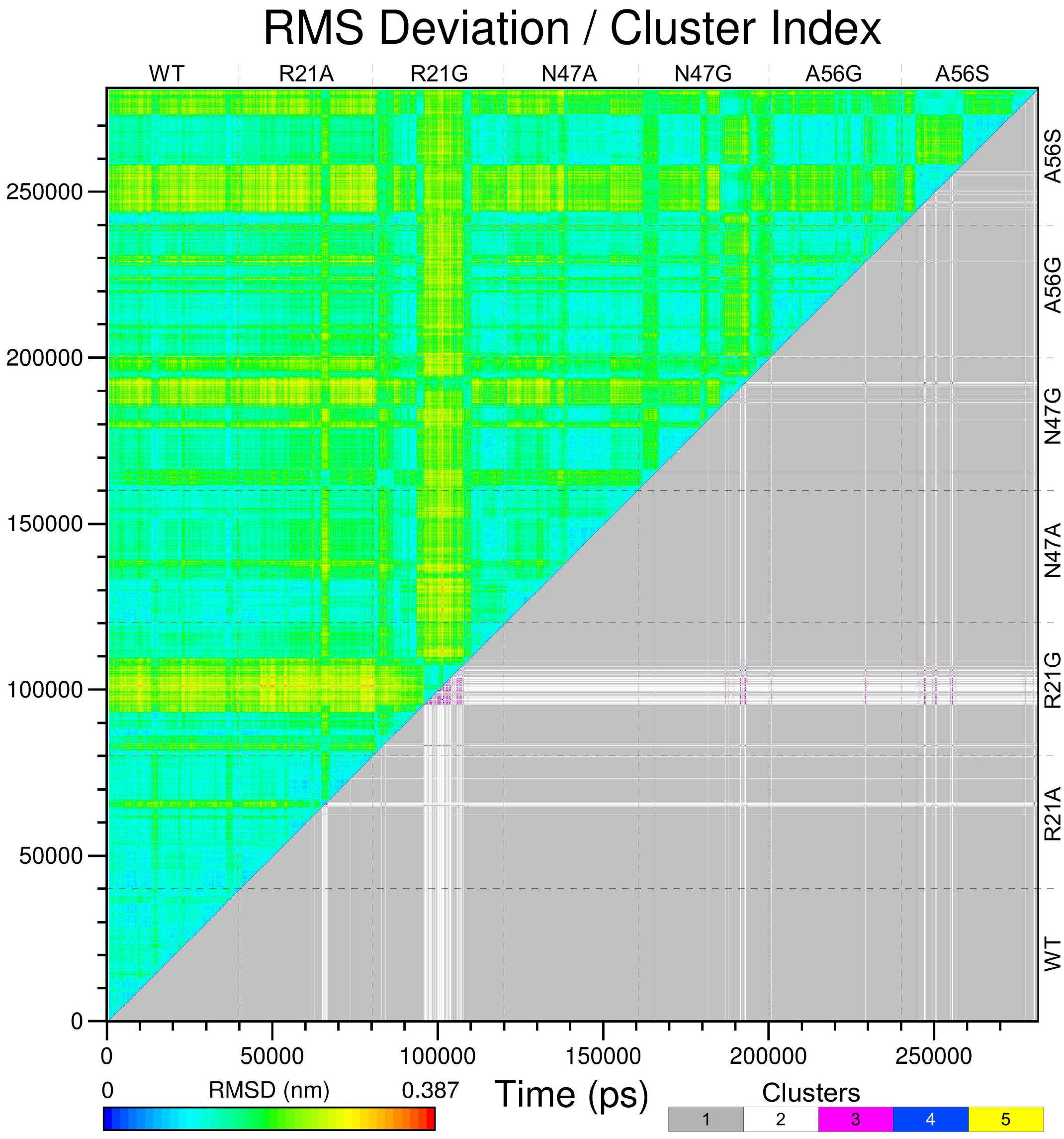

Supplement: Additional file 6 — Distance matrix and GROMOS clustering. The distance (RMSD) matrix for the combined trajectories of the ALL data set is reported in the upper triangular part of the image. The cluster attribution generated by GROMOS for a number of clusters equal to five is reported in the lower triangular part. [file 1471-2105-12-158-S6.PDF]
