# Supplementary material for: Conformational and functional analysis of molecular dynamics trajectories by Self-Organising Maps
Source: BMC Bioinformatics. 2011 May 14;12:158. doi: 10.1186/1471-2105-12-158 (PMC3118354; doi:10.1186/1471-2105-12-158)

Plot of RMSD versus time during the MD simulations.

From top to bottom, MD trajectories for the WT SH3, A56S and R21G mutants. Conformations attributed to the five clusters obtained from the SOM trained on the entire group of trajectories are coloured according to Figure 7.

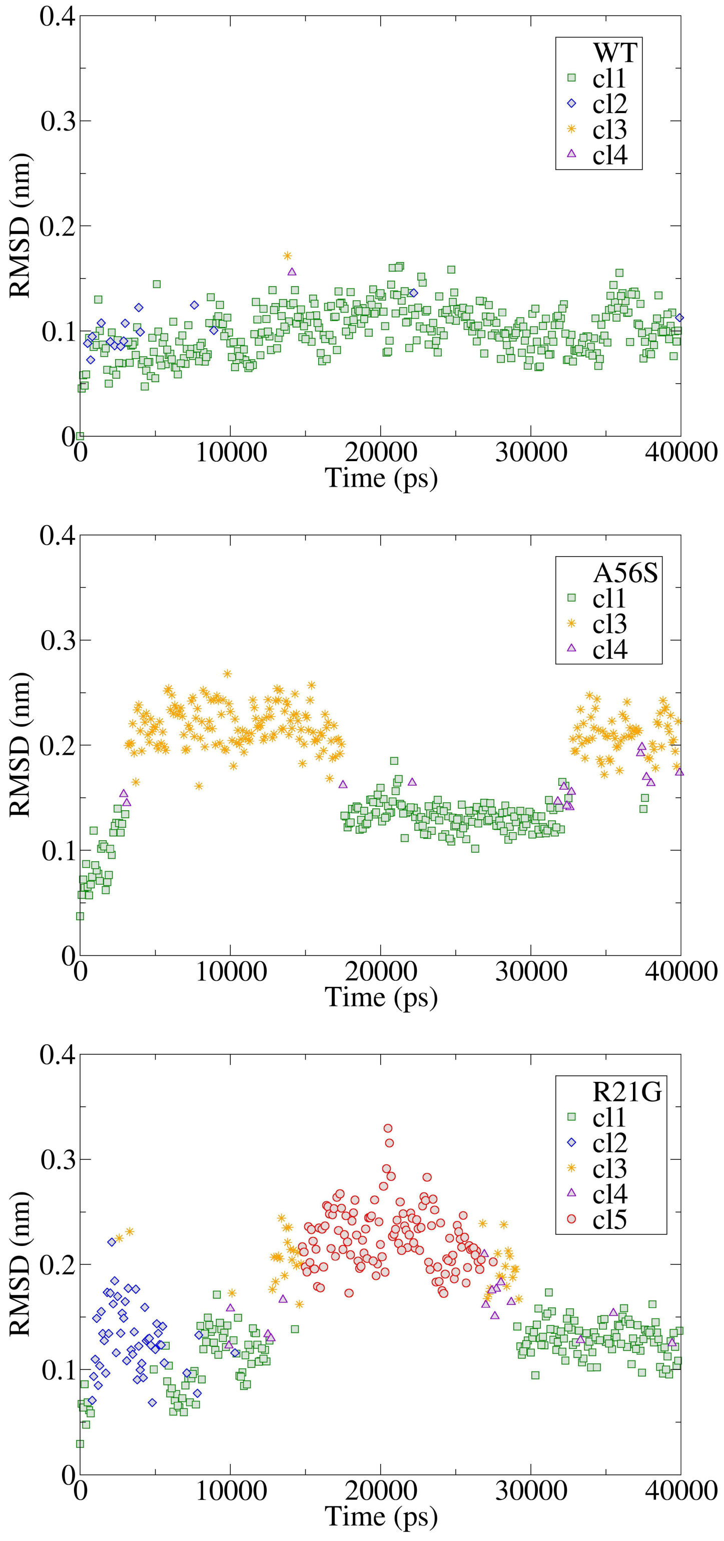

Supplement: Additional file 7 — Plot of RMSD versus time during the MD simulations. From top to bottom, MD trajectories for the WT SH3, A56S and R21G mutants. Conformations attributed to the five clusters obtained from the SOM trained on the entire group of trajectories are coloured according to Figure 7. [file 1471-2105-12-158-S7.PDF]
